# Indian Journal of Plastic Surgery in 2024

**DOI:** 10.1055/s-0045-1808251

**Published:** 2025-05-16

**Authors:** Maneesh Singhal, Shivangi Saha

**Affiliations:** 1 Department of Plastic, Reconstructive and Burns Surgery, All India Institute of Medical Sciences, Delhi, India

The *Indian Journal of Plastic Surgery (IJPS)* is increasingly becoming a reference journal in our field, not only in India but also in various countries across the globe. The journal has published several high-quality articles addressing multiple topics in the form of original investigations, reviews, case reports, letters, short communications, etc. This year we are looking forward to taking it to newer heights with the goal of continuing to contribute to the dissemination of high-quality content in plastic surgery. We took time to reflect on what the journal recently published so that we can continue to build upon the same.

In terms of journal metrics and outreach, the year 2023 saw a total of 307 submissions, with 91 articles accepted for publication. In 2024, with an increase in the number of issues, both submissions and acceptances rose significantly, reaching 424 submissions and 138 accepted articles. This reflected a rise in the acceptance rate from 29.6% to 32.6%.

Submissions were received from 34 countries, with the highest contributions coming from India, followed by Egypt, Mexico, Italy, the United Kingdom, and Indonesia. The submitted manuscripts comprised over 50% original articles, approximately 24 review articles, 12 systematic reviews, 228 letters to the editor, 20 innovation reports, and 130 case reports, etc.

The average review time was 34 days, a testament to the dedication and efficiency of our editorial board and reviewers. Notably, we expanded our panel by adding 150 accomplished expert reviewers—a significant milestone that has strengthened the peer review process and enhanced the journal's overall academic rigor. The journal published a wide array of articles, from complex randomized controlled trials and meta-analyses to special topics of interest to advanced case reports and series. In doing so, the journal touched on various areas of our plastic surgery domain, ranging from flaps, scar surgeries, and fat grafting to molecular studies. Although all the articles have their own merit and collectively contribute toward the dissemination of quality clinical information in your field, some of the articles stood out as notable mentions.

The journal published an engaging randomized clinical trial from the Iran University of Medical Sciences, addressing the aesthetic and functional outcomes of open carpal tunnel release and thread carpal tunnel release. 1 We published an original investigation exploring conversion to autologous breast reconstruction with latissimus dorsi and immediate fat grafting in patients with previous implant failure, from Universitario de Santiago de Compostela, Spain. 2 Further, the journal published a complex analytical article from Northwest University, Xi'an, Shaanxi Province, China, discussing in detail trends of research on platelet-rich fibrin. 3 Interestingly, the journal published a multicentric study, coming from various prestigious universities in Italy, discussing the risk of local breast cancer recurrence in implant-based breast reconstruction with macrotexturized and microtexturized prostheses. 4 We also published an engaging original investigation exploring the effect of basic fibroblast growth factors on perifascial areolar tissue transplants. 5 Rounding off our global authorship is an excellent meta-analysis discussing prospects for the use of botulinum toxin type A for the prevention of hypertrophic and keloid scars after surgeries coming from Saint Petersburg State Pediatric Medical University, Russia. 6

Our Indian authors contributed no less to the field than their overseas counterparts. The journal was enriched with some engaging topics that will draw the attention of national and global audiences alike. We published an innovative article bringing forth the concept of a novel asymmetric Y design of fascial sling for restoration of oral competence and adequate mouth opening in oral commissure defects following malignancy resection. 7 Exploring the oncology field, we published an interesting investigation discussing giant cell tumors of the tendon sheath of the hand, where the authors analyzed the factors impacting recurrence. 8 As a leading plastic surgery journal, it was only natural that we featured good-quality articles discussing various uses of flaps. These included articles that addressed flaps in different conditions ranging from chimeric anterolateral thigh flap (ALT) versus standard ALT flap in reconstruction of maxilloalveolar resections in head and neck cancers 9 to double V-Y flap in fingertip injury management. 10 We also had an overseas publication discussing the keystone design of the perforator flap. 11 Additionally, we published an interesting observation addressing the utility of fat grafting in chronic wounds. 12 Along similar lines, we had an original investigation exploring the aesthetic and psychological implications of hand rejuvenation by autologous fat grafting in post-Hansen's hand atrophy. 13

Apart from publishing engaging original articles and complex meta-analyses, we published interesting case series as well. One such study explored fish arrow injuries to the extremities. 14 Finally, the journal, keeping with the trend of the young generation, published a very interesting special topic addressing the concept of social media, where the authors discussed fantasies and fallacies of “aesthetic” Instagrammers. 15

We take great pride in what the journal has been publishing in the recent past and are motivated to carry the legacy forward. Our goal is to ensure that we publish high-quality articles addressing emerging topics that will educate the students and fascinate practicing clinicians alike. We take this opportunity to thank the esteemed reviewers of the journal, because of whom we have been able to publish such top-notch content. However, our deepest gratitude goes to the authors, who are the real reason why the *IJPS* has obtained such a respectful status among the medical and scientific fraternity, and we encourage them to continue enriching the journal with such engaging content.

## References

[1] Akhoondinasab M R Saraee A Akbari H Forghani S F Naderi B Aesthetic and functional outcomes of open carpal tunnel release and thread carpal tunnel release: a randomized clinical trial Indian J Plast Surg 2024 57 02 129 135 38774727 10.1055/s-0043-1778645 PMC11105821

[2] Couto-González I Brea-García B Fernández-Marcos AÁ Taboada-Suárez A Conversion to autologous breast reconstruction with latissimus dorsi and immediate fat grafting in patients with previous implant failure: an efficient, reproducible, and safe technique Indian J Plast Surg 2024 57 01 16 23 38450013 10.1055/s-0044-1779479 PMC10914543

[3] Zhao Y Dong C Fan L Focuses and trends of research on platelet-rich fibrin: a bibliometric and visual analysis Indian J Plast Surg 2024 57 05 356 363 39552803 10.1055/s-0044-1779478 PMC11567770

[4] Vinci V Klinger F Di Giuli R Breast cancer local recurrence risk in implant-based breast reconstruction with macrotexturized and microtexturized prosthesis: a multicentric retrospective cohort study Indian J Plast Surg 2024 57 05 372 378 39552806 10.1055/s-0044-1787059 PMC11567768

[5] Oshima J Shibuya Y Sasaki K Sekido M Effect of basic fibroblast growth factor in perifascial areolar tissue transplant Indian J Plast Surg 2024 57(1, suppl 1)S9 S15 39741735 10.1055/s-0044-1787561 PMC11684910

[6] Korableva N Romanenkov N Kremlev D Nekrasov A Miroshnichenko M Arbekov P Prospects for use of botulinum toxin type a for prevention of hypertrophic and keloid scars after surgeries Indian J Plast Surg 2024 57 06 421 431 39734381 10.1055/s-0044-1787175 PMC11679196

[7] Brajesh V Singh S Sarin D Novel asymmetric Y design of fascial sling for restoration of oral competence and adequate mouth opening in oral commissure defects post-malignancy resection Indian J Plast Surg 2024 57 02 147 151 38774734 10.1055/s-0043-1778643 PMC11105811

[8] Kolisetty P V Ali S S Ahmad I Sudhy I K Prakash O Kishore Y R Giant cell tumour a tumour of the tendon sheath of the hand: analysis of factors impacting recurrence Indian J Plast Surg 2024 57 02 123 128 38774731 10.1055/s-0044-1779657 PMC11105819

[9] Kumar V Jaffar S Mantri M Comparison of reconstruction of maxilloalveolar resections in head and neck cancers with chimeric anterolateral thigh flap (ALT) versus standard ALT flap Indian J Plast Surg 2024 57 03 173 178 39139681 10.1055/s-0044-1782200 PMC11319019

[10] Jayachandiran A P Rajendran S Ananthappan M Mahipathy S RRV Durairaj A R “Flap-in-flap” technique: double V-Y flap in fingertip injury management Indian J Plast Surg 2024 57 05 364 371 39552797 10.1055/s-0044-1787278 PMC11567765

[11] Bonetti C Arleo S Valdatta L Faini G The keystone design perforator flap: a flap to simplify complex reconstructive issues Indian J Plast Surg 2024 57(1, suppl 1)S30 S35 39741709 10.1055/s-0044-1788990 PMC11684907

[12] Prakash O Ali S S Yaseen M Sudhy I K Venkateshwar P K Kishore Y R Utility of fat grafting in chronic wounds Indian J Plast Surg 2024 57 03 201 207 39139688 10.1055/s-0044-1787174 PMC11319018

[13] Yamani V R Gurindagunta S V Raju R L Kumar S Valluri M K Sharma M Hand rejuvenation by autologous fat grafting in post-Hansen's hand atrophy: aesthetic and psychological implications Indian J Plast Surg 2024 57 06 461 468 39734368 10.1055/s-0044-1791943 PMC11679190

[14] Baba P UF Shah R A Yasir M Bashir S A Wani A H Fish arrow injuries to the extremities: a case series Indian J Plast Surg 2024 57 03 227 230 39139684 10.1055/s-0044-1787279 PMC11319010

[15] Sharma M Dhakad A Sharma S Fantasies and fallacies of “aesthetic” Instagrammers Indian J Plast Surg 2024 57 06 479 485 39734370 10.1055/s-0044-1790588 PMC11679195

